# Methicillin-resistant *Staphylococcus aureus* (MRSA) catheter-related bacteraemia in haemodialysis patients

**DOI:** 10.1186/s12879-015-1227-y

**Published:** 2015-10-30

**Authors:** Guillermo Cuervo, Mariana Camoez, Evelyn Shaw, María Ángeles Dominguez, Oriol Gasch, Belén Padilla, Vicente Pintado, Benito Almirante, José Molina, Francisco López-Medrano, Enrique Ruiz de Gopegui, José A. Martinez, Elena Bereciartua, Fernando Rodriguez-Lopez, Carlos Fernandez-Mazarrasa, Miguel Ángel Goenaga, Natividad Benito, Jesús Rodriguez-Baño, Elena Espejo, Miquel Pujol, On behalf of the REIPI/GEIH study group

**Affiliations:** Department of Infectious Diseases, Hospital Universitari de Bellvitge; Feixa Llarga s/n, 08907, L’Hospitalet de Llobregat, Barcelona, Spain; Department of Microbiology, H. Bellvitge, Barcelona, Spain; Department of Infectious Diseases, H. Parc Taulí, Sabadell, Spain; Department of Infectious Diseases, H. Gregorio Marañón, Madrid, Spain; Department of Infectious Diseases, H. Ramón y Cajal, Madrid, Spain; Department of Infectious Diseases, H. Vall d’Hebrón, Barcelona, Spain; Department of Infectious Diseases, H. Virgen del Rocío, Sevilla, Spain; Department of Infectious Diseases, H. 12 de Octubre, Madrid, Spain; Department of Microbiology, H. Son Espases, Palma de Mallorca, Spain; Department of Infectious Diseases, H. Clìnic, Barcelona, Spain; Department of Infectious Diseases, H. Cruces, Bilbao, Spain; Department of Infectious Diseases, H. Reina Sofía, Córdoba, Spain; Department of Microbiology, H. Marqués de Valdecilla, Santander, Spain; Department of Infectious Diseases, H. Donostia, Donostia, Spain; Department of Infectious Diseases, H. de la Santa Creu i Sant Pau, Barcelona, Spain; Department of Infectious Diseases, H. Virgen de Macarena, Sevilla, Spain; Department of Infectious Diseases, H. Terrassa, Terrassa, Spain

**Keywords:** Bacteraemia, Catheter-related, Haemodialysis, MRSA

## Abstract

**Background:**

The aim of the study was to determine clinical and microbiological differences between patients with methicillin-resistant S*taphylococcus aureus* (MRSA) catheter-related bacteraemia (CRB) undergoing or not undergoing haemodialysis, and to compare outcomes.

**Methods:**

Prospective multicentre study conducted at 21 Spanish hospitals of patients with MRSA bacteraemia diagnosed between June 2008 and December 2009. Patients with MRSA-CRB were selected. Data of patients on haemodialysis (HD-CRB) and those not on haemodialysis (non-HD-CRB) were compared.

**Results:**

Among 579 episodes of MRSA bacteraemia, 218 (37.7 %) were CRB. Thirty-four (15.6 %) were HD-CRB and 184 (84.4 %) non-HD-CRB. All HD-CRB patients acquired the infection at dialysis centres, while in 85.3 % of the non-HD-CRB group the infection was nosocomial (*p* < .001). There were no differences in age, gender or severity of bacteraemia (Pitt score); comorbidities (Charlson score ≥ 4) were higher in the HD-CRB group than in the non-HD-CRB group (73.5 % vs. 46.2 %, *p* = .003). Although there were no differences in VAN-MIC ≥1.5 mg/L according to microdilution, using the E-test a higher rate of VAN-MIC ≥1.5 mg/L was observed in HD-CRB than in non-HD-CRB patients (63.3 % vs. 44.1 %, *p* = .051). Vancomycin was more frequently administered in the HD-CRB group than in the non-HD-CRB group (82.3 % vs. 42.4 %, *p* = <.001) and therefore the appropriate empirical therapy was significantly higher in HD-CRB group (91.2 % vs. 73.9 %, *p* = .029). There were no differences with regard to catheter removal (79.4 % vs. 84.2 %, *p* = .555, respectively). No significant differences in mortality rate were observed between both groups (Overall mortality: 11.8 % vs. 27.2 %, *p* = .081, respectively), but there was a trend towards a higher recurrence rate in HD-CRB group (8.8 % vs. 2.2 %, *p* = .076).

**Conclusions:**

In our multicentre study, ambulatory patients in chronic haemodialysis represented a significant proportion of cases of MRSA catheter-related bacteraemia. Although haemodialysis patients with MRSA catheter-related bacteraemia had significantly more comorbidities and higher proportion of strains with reduced vancomycin susceptibility than non-haemodialysis patients, overall mortality between both groups was similar.

## Background

Methicillin-resistant *Staphylococcus aureus* (MRSA) bloodstream infection (BSI) has been a cause of major concern in healthcare systems all over the world, due to its high incidence rates and undesirable related outcomes [1–3]. Most series have found that the vascular catheter is the most frequent source of MRSA-BSI [2, 3].

Chronic haemodialysis patients, especially those dialysed through a venous catheter [4–6] are at a particularly high risk of this infection, with a rate more than 100 times higher than non-dialysis patients [4, 7]. Among haemodialysis patients with staphylococcal infections, those with MRSA infections have significantly longer hospitalizations, with higher costs, and are more likely to die within three months than those with methicillin-susceptible *Staphylococcus aureus* (MSSA) infections [8].

Patients with MRSA infections often receive antibiotic treatment with glycopeptides [9]. Some reports have raised concerns about the observation of higher vancomycin MICs in isolates of patients with MRSA bacteraemia who had received vancomycin within the last 30 days [10, 11], and other authors have also documented a significant risk for treatment failure and a higher mortality with increasing vancomycin MIC, even if MICs are in the susceptible range [12, 13]. Similar findings were reported specifically in patients undergoing haemodialysis, in whom a higher mortality and a longer mean hospital length of stay were also observed, with an ensuing increase in hospital costs [14].

Current information on this topic is still scarce in Europe, particularly in patients receiving haemodialysis who develop catheter-related MRSA bacteraemia (HD-CRB). We aimed to analyse this issue in a large multicentre prospective cohort of patients with MRSA bacteraemia from Spain. Our objective was to compare the clinical presentation, microbiological characteristics and outcomes in this group of patients and in patients with MRSA catheter-related bacteraemia who were not receiving HD (non-HD-CRB).

## Methods

### Study period and patients

This was post-hoc analysis of a prospective multicentre study of all consecutive episodes of MRSA bacteraemia in hospitalized adult patients occurring from June 2008 to December 2009 at 21 Spanish hospitals. The following information was collected from medical records using a standardized protocol: demographic characteristics, comorbidities, clinical features, antibiotic therapy, and outcomes. Patients with catheter-related bacteraemia (CRB) were selected for the study, and those undergoing haemodialysis (HD-CRB) were compared with those who were not (non-HD-CRB).

### Definitions

MRSA bacteraemia was defined as the presence of at least one positive blood culture for MRSA in a patient with clinical signs and symptoms of sepsis [15]. Vascular catheter-related bacteraemia was diagnosed using clinical and microbiological criteria defined by the guidelines of the Infectious Diseases Society of America [16]. It was considered when MRSA grew from at least one percutaneous blood sample culture and from a culture of the catheter tip, or when it grew in two blood samples for culture (one from a catheter hub and the other from a peripheral vein) that met criteria for quantitative blood cultures or differential time to positivity in a patient with accompanying clinical signs of sepsis and no other apparent source of infection. Complicated bacteraemia was defined as those episodes with positive follow-up blood cultures performed at 2–4 days after the beginning of adequate therapy and/or with evidence of metastatic infection or endocarditis [17]. Comorbidity was measured by the Charlson score, as described elsewhere [18]. Patients were classified into three categories on the McCabe and Jackson scale [19] according to their prognosis of survival before the MRSA bacteraemia: rapidly fatal (death expected within the following year), ultimately fatal (death expected within a period of 1 to 5 years), and non fatal (life expectancy of > 5 years). Severity of sepsis in the acute condition was assessed by the Pitt score [20]. Three acquisition categories were considered according to the Friedman criteria [21]: nosocomial, healthcare-related, and community-acquired bacteraemia. However, since all cases had healthcare exposure (i.e., dialysis) prior to culture, no community-associated cases were included. Distant extension was diagnosed in the presence of at least one distant infection secondary to blood spread seeding. The empirical antibiotic was defined as the antibiotic administered in the first 48 h after a positive blood culture was drawn, and it was considered appropriate if the strain was susceptible to at least one of the antibiotics administered according to the current CLSI breakpoints [22]. All patients were followed up to 4 weeks after completion of antibiotic treatment whether they were hospitalized or not. Recurrence within this period of time was defined as the isolation of MRSA in blood cultures after documented negative blood cultures or a newly diagnosed metastatic focus of the bacteremic past infection. Overall mortality was defined as death from any cause occurring in the 30 days and early mortality (EM) was defined for patients who died within the first two days after the onset of MRSA bacteraemia.

### Susceptibility testing and molecular epidemiology of MRSA isolates

MRSA strains were identified in each hospital, where preliminary susceptibility tests were performed. Isolates were then sent to a central reference laboratory. All *S. aureus* were identified by latex agglutination (Pastorex Staph-plus, Bio-Rad Laboratories, Madrid, Spain) and DNase production (DNasE-test Agar, BioMérieux, Marcy l’Etoile, France). Antimicrobial susceptibility was tested by the disc diffusion method according to the CLSI guidelines [22]. MICs were determined by the microdilution method in accordance with CLSI criteria by using commercial panels (ESTEN 2009, SensititreTM, Izasa, Barcelona, Spain) read visually. Vancomycin E-test (BioMérieux) MICs were determined using a 0.5 McFarland inoculum streaked evenly with a swab onto Mueller-Hinton agar plates [23]. PFGE was performed after SmaI restriction of chromosomal DNA [24]. Restriction patterns were interpreted in accordance with criteria published elsewhere [25]. Representative isolates of each PFGE type and subtype were studied to determine the Multilocus Sequence Type (MLST) [26] and the Staphylococcal Chromosome Cassette *mec* (SCC*mec*) types [27]. MLSTs and SCC*mec* types were further inferred for all the strains. The *agr* polymorphism and the presence of genes encoding class S (*lukS-PV*) and class F (lukF-PV) proteins for Panton–Valentine Leukocidin (PVL) were studied by PCR in all the isolates, following the methodology described elsewhere [28, 29].

### Statistical analysis

Continuous variables were compared using the Student’s *t*-test or the Mann–Whitney *U*-test as appropriate. Categorical data were compared using Fisher’s exact or Chi-squared tests. Analyses were performed using SPSS v15 (SPSS Inc., Chicago, IL, USA).

### Ethical considerations

This observational study was conducted in accordance with the Declaration of Helsinki and was approved by the Spanish Network for Research in Infectious Diseases (REIPI) and by the Institutional Review Board at each participating centre: H. Arnau de Vilanova, Lleida; H. Universitari de Bellvitge, Barcelona; H. de Burgos, Burgos; H. Clìnic, Barcelona; H. Universitario 12 de Octubre, Madrid; H. de Cruces, Barakaldo; H. de Donostia, Donostia; H. General Universitario Gregorio Marañón, Madrid; H. Universitari Joan XXIII, Tarragona; H. Universitari del Mar, Barcelona; H. Universitario Marques de Valdecilla, Santander; H. Universitari Mutua de Terrassa, Terrassa; H. del Parc Taulí, Sabadell; H. Universitario Ramon y Cajal, Madrid; H. Universitario Reina Sofía, Córdoba; H. San Pedro de la Rioja, Logroño; H. de la Santa Creu i Sant Pau, Barcelona; H. Universitari Son Espases, Palma de Mallorca; H. Universitario Virgen Macarena, Sevilla; H. Universitario Virgen del Rocío, Sevilla; H. Universitari Vall d’Hebrón, Barcelona. To protect personal privacy, identifying information of each patient in the electronic database was encrypted. Informed consent was waived by the Clinical Research Ethics Committee because no intervention was involved and no information able to identify the patient was included.

## Results

### Characteristics of patients

**Fig. 1:**
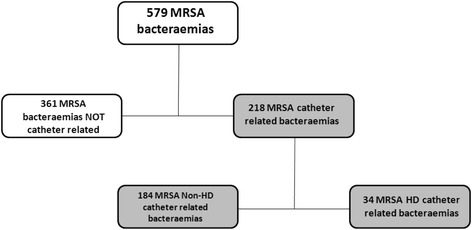
Flow chart of patients included in analysis

From a total of 579 episodes of MRSA bacteraemia, 218 (37.7 %) were catheter-related (Fig. 1): 34 (15.6 %) occurred in the HD-CRB group and 184 (84.4 %) in the non-HD-CRB group. The clinical and microbiological characteristics of patients are shown in Table 1. The comparison between the groups did not find significant differences in gender or age distribution. All HD-CRB patients acquired the infection at dialysis centres (chronic ambulatory haemodialysis), while the acquisition in non-HD-CRB patients was nosocomial in 85.3 % of cases (*p* < .001). Comorbidities measured by the Charlson score were higher in the HD-CRB group (Charlson ≥4: *n* = 25, 73.5 % vs. *n* = 85, 46.2 %, *p* = .003) while the severity of the underlying disease according to the McCabe scale was lower in this group (McCabe ≥2: *n* = 13, 38.2 % vs. *n* = 97, 52.7 %, *p* = .120). No differences were observed between groups in the severity of bacteraemia measured by the Pitt score.

**Table 1:** Clinical and microbiological characteristics of patients by group

	HD-CRB *n* = 34 (100 %)	Non-HD-CRB *n* = 184 (100 %)	*p*
Male Sex	23 (67.6)	113 (61.4)	.491
Age > 70 y	16 (47.1)	92 (50)	.753
Acquisition			
Health-care	34 (100)	27 (14.7)	<.001
Nosocomial	0	157 (85.3)	<.001
Charlson score > 4	25 (73.5)	85 (46.2)	.003
McCabe scale > 2	13 (38.2)	97 (52.7)	.120
Pitt score > 3	6 (17.6)	55 (29.9)	.144
Microbiology			
agr type^a^ I	3 (10)	44 (24.9)	.082
II	27 (90)	132 (74.6)	.354
PFGE type^a^ 2	25 (83.3)	107 (60.5)	.091
4	2 (6.7)	18 (10.2)	.688
Other	3 (10)	52(29.3)	.046
Clonal Complex^b^			
5	28 (93.3)	128 (74.4)	.128
8	0	14 (8.1)	.199
22	2 (6.7)	17 (9.9)	.759
Other	0	13 (7.6)	.774
PVL^a^	0	2 (1.1)	1
Microdilution Vancomycin MIC^a^ > = 1.5 mg/L	0	(4.9)	.363
E-test Vancomycin MIC^a^ > = 1.5 mg/L	19 (63.3)	78 (44.1)	.051

^a^Data available in 207 patients

^b^Data available in 202 patients. Within Clonal Complex 5, 124 isolates belonged to the ST125 clone (HD-CRB: 25/83.3 % vs. Non-HD-CRB: 99/57.6 %; *p* = .013) whereas 9 isolates belonged to the ST146 clone (HD-CRB: 2/6.7 % vs. Non-HD-CRB: 7/4 %; *p* = .875)

Abbreviations: *HD-CRB* Haemodialysis catheter-related bacteraemia, *Non-HD-CRB* Non-haemodialysis catheter-related bacteraemia, *agr* Accessory gene regulator, *PFGE* Pulsed field gel electrophoresis, *PVL* Panton Valentine Leukocidin, *MIC* Minimum inhibitory concentration

### Microbiologic studies

A dominant PFGE genotype (pulse-type 2) was found in both groups (Table 1), with all isolates belonging to Clonal Complex (CC) 5 (ST125 and ST146). They carried a SCC*mec* element type IV and *agr* type 2. PVL was positive in only two isolates in the non-HD-CRB group, both belonging to the USA300 clone. No isolates had vancomycin MIC ≥1.5 mg/L tested with the broth microdilution method in the HD-CRB group, compared with seven isolates (5 %) in the non-HD-CRB group (*p* = .60). However, when MIC was assessed by the E-test, 19 patients (63.3 %) in the HD-CRB group had vancomycin MIC ≥1.5 mg/L, and 78 (44.1 %) in the non-HD-CRB (*p* = .051).

### Treatment and outcomes

The treatment and clinical outcomes are detailed in Table 2. Empirical therapy was considered appropriate in 31 patients (91.2 %) in the HD-CRB group vs. 118 (64.1 %) in the non-HD-CRB group (*p* = .001). Vancomycin was the most frequent empirical therapy in the HD-CRB group, in which 28 patients received vancomycin treatment (82.4 %) versus 78 patients (42.4 %) in the non-HD-CRB group (*p* = <.001). There were no significant differences between groups in the number of catheters removed during the episode (*n* = 27, 79.4 % vs. *n* = 155, 84.2 %, *p* = .555). Although no significant differences in outcomes were observed, there was a trend towards recurrent bacteraemia in the HD-CRB group (3/34, 8.8 % vs. *n* = 4/184, 2.2 %, *p* = .076).

**Table 2:** Treatments and clinical outcomes of patients by group

	HD-CRB *n* = 34 (100 %)	Non-HD-CRB *n* = 184 (100 %)	*p*
Treatment (within 48 h)			
Catheter removal	27 (79.4)	155 (84.2)	.555
Vancomycin therapy	28 (82.3)	78 (42.4)	<.001
Appropriate ATB therapy^a^	31 (91.2)	118 (64.1)	.002
Outcome			
Persistent bacteraemia 3d	5 (14.7)	37 (20.1)	.463
Persistent bacteraemia 7d	2 (5.9)	26 (14.1)	.533
Complicated bacteraemia	11 (32.4)	89 (48.4)	.095
Distant secondary focus	5 (15.2)	38 (20.7)	.465
Endocarditis	0	10 (5.4)	.368
Recurrence	3 (8.8)	4 (2.2)	.076
Early mortality (48 h)	0	7 (3.8)	.599
Overall mortality (30d)	4 (11.8)	49 (27.2)	.081

^a^ATB therapy: Antibiotic therapy

## Discussion

In this multicentre study of a large Spanish cohort including a high number of patients with MRSA catheter-related bacteraemia, patients receiving haemodialysis did not present worse outcomes than the other group of patients with MRSA catheter-related bacteraemia. These findings challenge the results of previous reports which suggested that patients receiving HD are more prone to complications and poor prognosis [30, 31].

It is known that HD patients are frequently and recurrently colonized by MRSA [32, 33]. In fact, this pathological condition is a well known risk factor for MRSA bacteraemia [4, 34]. This is a matter of concern, as the size of the dialysis population is increasing worldwide [35], as well as the proportion of HD patients using a catheter [36].

In our cohort of 579 MRSA patients, 218 (37.7 %) had catheter-related bacteraemia, a rate similar to other reports [37]. Patients on HD through a catheter had a greater burden of comorbidities as measured by Charlson score, a frequent finding in these patients [38].

Regarding vancomycin susceptibility tests, our findings reproduce the highly variable and method-dependent results already reported by some authors [39, 40]. None of our HD-CRB patients had high MICs according to microdilution, but the E-test method identified high MICs in 19 of them. In spite of these E-test results, however, our HD patients did not have poorer outcomes, in agreement with other reports [14, 41]. In other words, the MIC measured by E-test did not improve the predictive ability of the microdilution in our cohort, which challenges us about its accuracy as a prognostic reference method. Vancomycin was the most frequently prescribed empirical therapy in our HD patients; its administration certainly is a common practice in this population [9]. The high presumption of MRSA in HD patients with a suspected infection may explain the choice of a more appropriate antibiotic therapy for this group both in our cohort and in others [42].

Although no significant differences in outcomes between groups were observed, HD patients had a tendency towards a higher recurrence rate, in agreement with previous research [43] but not a higher frequency of endocarditis [5]. Taken together, patients with MRSA catheter-related bacteraemia in our cohort had lower early and overall mortality rates (3.2 % and 24 % respectively) compared with studies which analysed MRSA bacteraemia of all sources [44]. In fact, patients with catheter-related bacteraemia represent a particular group in which the main therapeutic strategy is catheter removal; this was achieved within 48 h in 80 % of our patients. Furthermore, some authors have suggested that, rather than vancomycin MIC, the anatomical site of infection may be the best predictor of therapy success [42]. Finally, our HD patients exhibited a trend towards lower early and total mortality compared with the other group in which there was significantly more inappropriate initial treatment, a well known predictor of mortality [45]. In agreement with our findings, some recent reports on *Staphylococcus aureus* bacteraemia (both MSSA and MRSA) found lower 30 and 90 days case fatality rates in patients with end stage renal disease, irrespective of the type of replacement therapy received [46].

Our study has some limitations. First, it included a relatively low number of HD patients with CRB, and its multicentre nature may have introduced some differences between centres regarding the clinical management. However, to our knowledge, it is the first study to address this specific population group within all patients with MRSA bacteraemia. On the other hand, isolates were frozen prior to MIC testing, which potentially could have underestimated MIC lecture in both groups [47].

## Conclusion

In our multicentre study, ambulatory patients in chronic haemodialysis represented a significant proportion of cases of MRSA catheter-related bacteraemia. Although haemodialysis patients had significantly more comorbidities and higher proportion of strains with reduced vancomycin susceptibility than non-haemodialysis patients, they do not have worse outcomes. In fact, the higher frequency of appropriate empirical antimicrobial therapy may explain the trend towards better outcomes in this group, even though the sample size could prevent its statistical confirmation.
